# Kikuchi-Fujimoto disease presenting with prolonged fever and aseptic meningitis in a child: a case report

**DOI:** 10.3389/fped.2025.1572816

**Published:** 2025-05-08

**Authors:** Panying Li, Jing Yang, Li Mao, Li Huang, Qian Ni

**Affiliations:** ^1^ The Second Clinical Medical College, Lanzhou University, Lanzhou, China; ^2^ Pediatric Nephrology Department, The Second Hospital of Lanzhou University, Lanzhou, China; ^3^ Pediatric Respiratory Department, The Second Hospital of Lanzhou University, Lanzhou, China

**Keywords:** prolonged fever, aseptic meningitis, kikuchi-Fujimoto disease, lymphadenopathy, child

## Abstract

Kikuchi-Fujimoto Disease (KFD) is a rare, self-limiting lymphadenitis that predominantly affects young women of Asian descent and is less frequently encountered in children. The disease is characterized by focal and indurated cervical lymphadenopathy with fever and other infrequent systemic manifestations, including neurologic symptoms that are rare. This report details the diagnosis and treatment of a 14-year-old male with an atypical case of KFD. He exhibited a fever that persisted for over 1 month together with dizziness, nausea, arthralgia, night sweats, weight loss, and splenomegaly. On day 16 following fever onset, he presented with symptoms of aseptic meningitis, with symptoms of cervical lymph node swelling and pain only manifesting on day 25 after fever onset. Positron emission tomography (18F-FDG PET/CT) revealed the enlargement of lymph nodes in several regions of the body. After considering a diagnosis of lymphoma, KFD was ultimately diagnosed via cervical lymph node biopsy. His condition improved following oral prednisone administration. This case report highlights the complex disease course of KFD and the difficulties associated with diagnosing it at an early stage. KFD is rarely considered in the differential diagnosis for children with prolonged unexplained fever, especially with delayed lymphadenopathy, leading to potential misdiagnosis and unnecessary investigations.

## 1 Introduction

Kikuchi-Fujimoto Disease (KFD), also known as histiocytic necrotizing lymphadenitis or subacute necrotizing lymphadenitis, is a rare, benign disease of the lymph nodes that was first reported in 1972 by the Japanese pathologists Kikuchi and Fujimoto (1, 2). KFD is most frequently diagnosed in young Asian women, and its overall incidence remains uncertain, with cases in children being rare and likely underdiagnosed (3).

The first symptom of KFD in many patients is cervical lymph node enlargement, together with fever in 30%–50% of cases. Patients can also experience a range of less common systemic symptoms such as fatigue, weight loss, night sweats, arthralgia, rash, and hepatosplenomegaly (4–6). Neurological complications are even rarer and most frequently include aseptic meningitis or aseptic encephalitis (7).

Given its variable and nonspecific clinical manifestations, distinguishing between KFD and infectious, malignant, or autoimmune diseases remains challenging, particularly during the early stages of the disease, with a high risk of missed diagnosis or misdiagnosis. In this report, we describe a case of atypical KFD in a child, with the aim of raising awareness of this disease among pediatricians.

## 2 Case presentation

A 14-year-old male presented to our outpatient clinic with fever that had persisted for 8 days with no obvious cause, recurring 2–3 times per day with peak temperatures of 39.3°C. The fever was accompanied by nausea, dizziness, and occasional generalized joint pain, primarily involving the bilateral hand and knee joints. His body temperature did not return to normal after administration of oral ibuprofen and a Pudilan anti-inflammatory oral solution. Outpatient examinations showed that rheumatoid factor was negative, while the levels of inflammatory markers were significantly elevated (Table 1). The patient was treated with intravenous amoxicillin for 4 days at a local hospital but the fever did not resolve and he thus returned to our outpatient clinic and was admitted to hospital with “fever to be investigated”. The patient had not exhibited any respiratory or gastrointestinal symptoms since the onset of the illness, nor had he experienced headaches or other neurological symptoms. His past medical history and family history were unremarkable. He also had no recent history of travel, cat bites/scratches, or exposure to patients with tuberculosis.

**Table 1:** Laboratory test results.

Parameter	Day 8	Day 13	Day 17	Day 23	Day 25	Day 36	Reference range
White blood cell count（10^9^/L）	5.1	9.5	3.3	3.23	2.38	3.35	4.6–11.3
Neutrophil ratio（%）	0.53	0.78	0.44	0.35	0.45	0.33	0.33–0.74
Lymphocyte ratio (%)	0.37	0.16	0.46	0.52	0.50	0.57	0.20–0.54
Hemoglobin（g/L）	137	149	136	136	141	137	131–179
Platelets（10^9^/L）	295	272	314	313	320	310	148–399
C-reactive protein (mg/L)	63.79	57.71	26.71	28.14	27.25		<10
Ferritin（ng/L）		297			731		30–400
Amyloid A (mg/L)	33.76	32.33			121		<10
Anti-streptococcal hemolysin (IU/ml)	164.50	170.40		180.70			<150.00
Erythrocyte sedimentation rate (mm/h)		29.0			30.0		<10
Calcitoninogen (ng/ml)		0.079	0.068				0.000–0.046
Lactate dehydrogenase (U/L)		334				247	120–250

Examination on admission indicated that the patient was alert but fatigued, with no evidence of a generalized rash or palpable superficial lymph node involvement. Mild pharyngeal congestion was observed, and the tonsils were enlarged by 1 degree, with white secretions being evident on the tonsillar surface. His neck was soft and non-resistant. Auscultation of both lungs revealed coarse respiratory sounds without wet rhonchi. The liver and spleen were not palpable under the ribs, nor was there any evidence of joint redness, swelling, or tenderness. The neurological findings, including meningeal signs, were unremarkable.

The laboratory results from this patient following admission are presented in Table 1. Blood culture, mid-stage urine culture, throat swab culture, respiratory pathogens, Epstein–Barr virus, tuberculosis, and infectious disease-related test results were all negative. The patient was also negative for IgM antibodies specific for Coxsackie group B viruses, while his IgG results were positive. The test results for immunoglobulin, anti-nuclear antibodies (ANA), anti-neutrophil cytoplasmic antibodies (ANCA), anti-double-stranded DNA antibodies (dsDNA), lymphocyte subpopulation analysis, and human leukocyte antigen B27 nucleic acid were all negative, and peripheral blood and bone marrow smear results were both normal.Chest CT scans revealed uneven lung translucency bilaterally and thickened bronchial walls, while x-ray radiography of the long bones of the limbs, abdominal ultrasonography, and vascular ultrasound imaging of the lower limbs failed to reveal any abnormalities.

**Figure 1:**
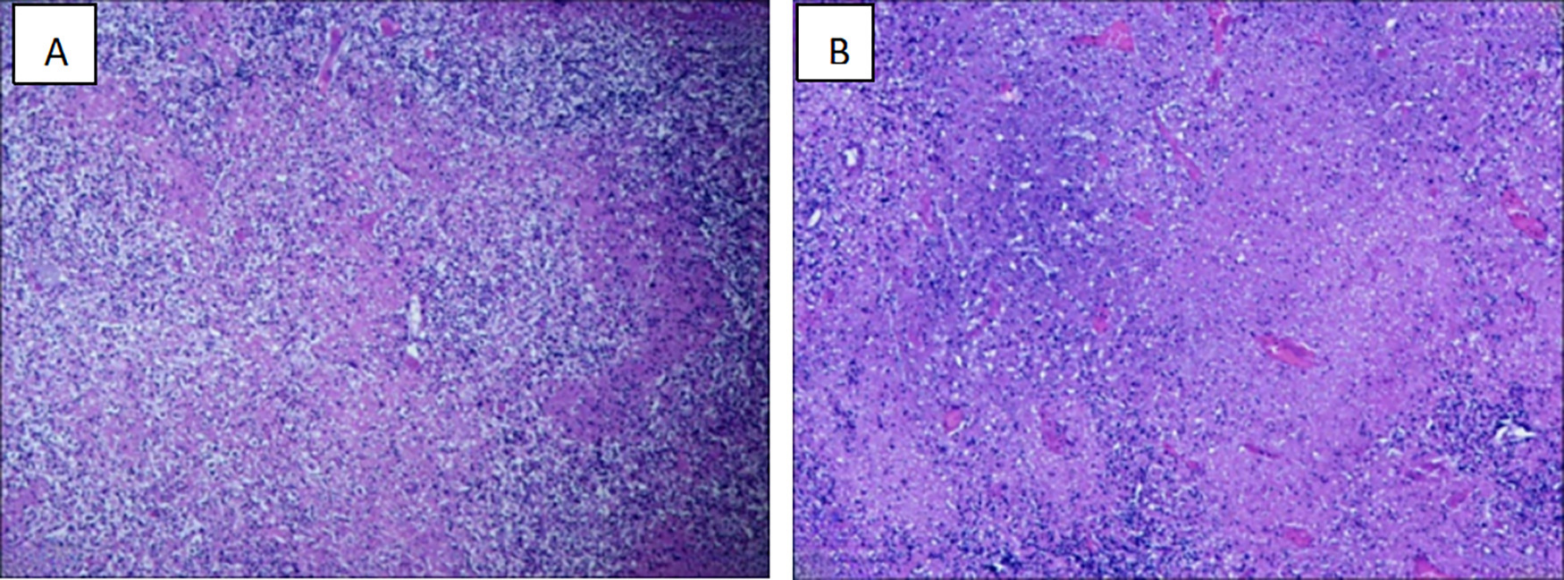
Histopathology of cervical lymph nodes consistent with the diagnosis of KFD. **(A)** Multiple irregularly shaped necrotic areas were seen in the lymph node (H&E, × 200). **(B)** Multiple histiocytosis around the necrotic foci, with a large number of fragmented nuclei, multifocal hemorrhages, and no clear neutrophils (H&E, × 400).

Based on these signs, symptoms, and laboratory test results, the child was preliminarily diagnosed with acute purulent tonsillitis, and he was treated with cefazolin sodium for 3 days. However, he continued to experience a recurrent fever 2–3 times per day with a maximum temperature of 39.2°C. On day 16 after fever onset, the child developed a headache and vomited twice. A lumbar puncture was performed, and the resultant cerebrospinal fluid examination results are presented in Table 2. No obvious abnormalities were detected through cranial nuclear magnetic resonance imaging (MRI) or electroencephalogram examination. The child was thus diagnosed with aseptic meningitis and received symptomatic treatment aimed at lowering intracranial pressure levels, which successfully relieved his symptoms. On day 17 after fever onset, reexamination of his blood routine leukocyte counts revealed that their levels were reduced (3.3 × 10^9^/L), and lower inflammatory biomarker levels were evident as compared to prior examination (Table 1). His peak fever level also began to decline, and the interval between fevers grew increasingly prolonged. On day 20 after fever onset, his temperature had returned to normal. After a further 2-day interval, the child experienced another bout of pyrexia occurring 1–2 times per day with a peak temperature of 38.6°C. Enhanced chest and abdominal CT scans did not reveal any obvious chest abnormalities, but multiple lymph nodes in the mesenteric region of the right middle and lower abdomen were enlarged, and blurring of the adjacent fat interstitial space was observed, while the presence of inflammatory lesions could not be ruled out. On day 25 after fever onset, the patient developed bilateral neck pain together with pronounced fatigue and night sweats. 18F-FDG PET/CT imaging revealed multiple enlarged lymph nodes with increased FDG metabolism in the bilateral parapharyngeal space, neck, clavicular region, right axilla, hepatoportal region, abdominal cavity, retroperitoneum, right pelvic wall, and right inguinal region, with the increase FDG metabolism suggesting the potential for lymphoma such that biopsy was suggested for clarification. In addition, slight splenic enlargement was observed, with diffuse, mildly increased FDG metabolism, and the potential for functional reactive enhancement was considered. Pathological examination following the biopsy of the left cervical lymph node revealed lymph node necrosis with abundant histiocytes surrounding proliferating necrotic foci and the absence of any neutrophils, consistent with the presentation of KFD (Figure 1). At this point, in addition to the other symptoms discussed above, the patient had lost approximately 10 kg of weight.

**Table 2:** Lumbar puncture results.

Item	Day 16	Day 23	Reference value
Pressure（mmH_2_O）	140	153	80∼180
Appearance	Colorless and transparent	Colorless and transparent	Colorless and transparent
Pandy's test	Negative	Weakly positive	Negative
White blood cell count (10^6^/L)	26	8	0∼15
Percentage of mononuclear cells (%)	92	0	
Percentage of multinucleated cells (%)	8	0	
Protein（g/L）	0.29	0.29	0.20∼0.40
Glucose (mmol/L)	2.3	2.6	2.5∼4.4
Chloride (mmol/L)	119.5	117.9	120.0∼130.0
General bacterial culture	No bacterial growth		Negative
India ink staining	Negative		Negative
Rapid test for nucleic acid of tubercle bacilli	Negative		Negative
Herpes simplex virus type II quantitative nucleic acid PCR test	Negative		Negative
Metagenomic next-generation sequencing	Negative		

Pandy's test: used to detect the elevated levels of protein in cerebrospinal fluid; India ink staining: For the detection of Cryptococcus in cerebrospinal fluid; Metagenomic next-generation sequencing: Using high-throughput sequencing technology to non-targetedly detect the nucleic acids of bacteria, fungi, viruses, parasites, and other pathogens present in cerebrospinal fluid.

Following diagnostic confirmation, the patient was treated with oral prednisone (1 mg/kg/day), and his temperature returned to normal levels after 2 days, gradually withdrawing prednisone over the course of 3 months. The child was asymptomatic, autoantibody-negative, and in generally good condition on 6-month follow-up.

## 3 Discussion

KFD is a benign, self-limiting form of necrotizing lymphadenitis that is most common among Asian women under 40 years of age, although it can affect patients of any age or ethnicity, with peak incidence between the ages of 25 and 29 (8). Cases of KFD in children have only rarely been reported, with most affecting children over the age of 10 and with a male-to-female ratio of 1.2:1 (9).

The onset of KFD can be acute or subacute, with the disease progressing over a period of weeks. The first symptom is generally, cervical lymphadenopathy, with unilateral posterior cervical lymph node involvement in 60%–90% of cases, while the enlargement of the bilateral cervical lymph nodes is comparatively rarer, and 1%–22% of cases present with generalized lymphadenopathy.Nodal enlargement in affected patients often coincides with acute or dull pain, although painless enlargement is evident in 40% of cases (10). A fever affects 30%–50% of patients with KFD, most often in the form of a low-grade intermittent fever lasting for roughly a week, although in rarer cases it can persist for up to a month (4, 5). In a recent study of 114 children with KFD, however, 62% of these children presented with a high fever (>39°C), and 44% experienced a persistent fever lasting for more than 14 days (11). Other uncommon KFD-related symptoms affecting under 5% of patients can include malaise, weight loss, sore throat, rash, arthralgia, upper respiratory symptoms, night sweats, splenomegaly, and hepatomegaly (5). Central nervous system (CNS) involvement is rare in patients with KFD, with only a few reports of patients exhibiting aseptic meningitis, encephalitis, optic neuritis, cerebellar ataxia, or hemiparesis. The most common of these neurological complications is aseptic meningitis, which affects 2.8%–9.8% of patients with KFD (7), resulting in symptoms including vomiting, headache, and convulsions. The onset of meningitis generally occurs 2–3 weeks following lymphadenopathy, although these neurologic symptoms can rarely precede any apparent lymphadenopathy (7, 12, 13). In the present case, the patient's first symptom was a persistent unexplained fever of up to 39.3°C. In addition to presenting with rare systemic symptoms including nausea, dizziness, night sweats, weight loss, and splenomegaly, this patient developed aseptic meningitis before the appearance of swollen and painful lymph nodes. He first presented with swollen, painful cervical lymph nodes 25 days following fever onset, and he exhibited extensive nodal involvement. This case underscores the potential complexity of the clinical course of KFD, which is rarely considered in the differential diagnosis of patients affected by delayed lymphadenopathy, even though it can be a cause of unexplained febrile illness.

Laboratory findings can be unremarkable in most patients with KFD and are therefore not recommended for the diagnosis of this condition, but are nevertheless important for ruling out other diseases. Common findings in patients with KFD can include leukopenia in routine blood tests and the presence of atypical lymphocytes in peripheral blood smears. Mildly elevated levels of peripheral inflammatory biomarkers including C-reactive protein, erythrocyte sedimentation rate, and calcitoninogen may also be observed, together with other nonspecific manifestations including elevated serum lactate dehydrogenase and ferritin levels. Thrombocytopenia, pancytopenia, and anemia are rarely reported (4, 5, 14). In patients with KFD who have aseptic meningitis, cerebrospinal fluid analyses tend to be negative for any bacteriological cultures, with no evidence of pressure abnormalities, chloride and glucose levels within the reference range, and leukocyte levels that are normal or only slightly elevated (12). In children with KFD, a high fever (≥39.0°C), generalized lymphadenopathy, splenomegaly, multiple organ involvement, leukopenia, anemia, and elevated levels of liver enzymes, ferritin, and inflammatory markers are positively correlated with disease course and patient condition (14), and the presentation of the child in this report was consistent with this observation.

KFD is not characterized by any distinctive imaging findings that can aid in patient diagnosis, and while ultrasound examination of the cervical lymph nodes or cervical CT or MRI imaging can be conducted, it can be challenging to differentiate between KFD and other diseases of the lymph nodes.18F-FDG PET/CT offers greater diagnostic utility for affected patients, with most such analyses having been performed owing to the resemblance of KFD to symptoms of malignant disease in some patients. However, these PET/CT findings can be readily confused with malignant lymphoma, underscoring a need for caution when interpreting the results. PET/CT analyses of patients with KFD have revealed the presence of multiple hypermetabolic lymph nodes, even though a few of these nodes were enlarged. Enhanced bone marrow and splenic metabolism has been reported in some cases, albeit without any liver abnormalities (15, 16). In line with the findings in the present case, Kim et al. reported that patients with malignant lymphoma often present with high levels of FDG uptake, extranodal involvement, or large mass lymphadenopathy upon PET/CT examination. In contrast, patients with KFD generally only exhibit localized FDG uptake, generalized lymphadenopathy, and the absence of extranodal involvement, providing an opportunity to differentiate between these conditions (16, 17).

Lymph node biopsy is currently required to definitively diagnose KFD. Relative to excisional lymph node biopsy, fine-needle aspiration cytology is a safer and simpler approach for children, although its accuracy is only 56.3% in KFD cases, such that it is a poor alternative to lymph node biopsy (4, 18). In some reports, rapid improvements in patient condition have been described following excisional lymph node biopsy, suggesting that diseased lymph node removal may offer both diagnostic and therapeutic utility (14, 19). Short-term oral glucocorticoid administration is the most common approach to treating patients suffering from prolonged or severe disease. While earlier studies cited recurrence of KFD at 3% to 4%, a recent study of children with KFD reported its recurrence rate at approximately 42% (20). Recurrence is more frequent in children with other immunologic diseases. Therefore, long-term follow-up of children diagnosed with KFD is recommended.

## 4 Conclusions

KFD is characterized by varying clinical manifestations such that lymphadenopathy may not be the first or most prominent symptom in children, leading to a high risk of the disease being overlooked, subjecting patients to unhelphul investigations and treatments.

This case report underscores the need for clinicians to consider KFD in the differential diagnosis of patients suffering from prolonged unexplained fever. In these cases of prolonged fever, lymph node-related analyses should be conducted as quickly as possible. In addition to the advantages of the ultrasound-based evaluation of the cervical lymph nodes, PET/CT imaging can also provide diagnostic advantages, although pathologic examination is ultimately necessary to achieve a final confirmatory diagnosis.

## Data Availability

The original contributions presented in the study are included in the article/Supplementary Material, further inquiries can be directed to the corresponding author.
